# An Inquiry into the Present State of Medical Surgery; Including the Analogy Betwixt External and Internal Disorders; and the Inseparability of These Branches of the Same Profession

**Published:** 1784

**Authors:** 


VI. *An Inquiry into the prefent State of Medical Surgery; including the analogy betwixt external and internal diforders; and the infeparability of thefe branches of the fame profeffion. By* Thomas Kirkland, M. D. *Member of the Royal Medical Society at Edinburgh.* Vol. I. 8vo. Dodfley, *London.* 500 pages.

IN this inquiry into the prefent ftate of medical furgery, the profeffed aim of Dr. Kirkland is to encourage the ftudy of a branch of the profeffion which he thinks has hitherto not been duly attended to by modern writers.

An Effay on the infeparability of the different branches of phyfic is prefixed to the work, by way of introduction; and this is followed by a differtation on the brain and nerves, and another on the fympathy of the nerves, prefixed to the author's treatife on the child-bed fever, and here re-printed, with additions; after which he treats of irritability in general; of the the pulse; and of the nature and cure of fevers. His observations on the last of these subjects are comprised in a short practical abstract of a work he formerly published, on fevers.

The remainder of the volume consists of a treatise on inflammation, and its consequences.

Dr. Kirkland begins this part of his work with remarks on inflammation in general; after which he considers the manner in which nature herself terminates different kinds of inflammation, and then proceeds to treat, in the following order, of a simple inflammation of the skin—erysipelas in general, and its varieties—cure of erysipelas—local erysipelas—critical erysipelata, including those occasioned by contagious miasmata—inflammatory and nervous rheumatism—phlegmonoide rheumatism—gout—inflammatory oedema—and lastly, ophthalmia.

Of the plan and merits of this performance, as a systematic work, no precise idea can be formed, till the whole is published. Whether it is to be completed in one or more volumes, the author has omitted to inform us.

To the author's opinion concerning the inseparability of the different branches of physic, as well as to ſome points of theory in the preſent volume, objections might, perhaps, with good reaſon be made; but ſuch objections would appear trifling to the practical reader, who will find interſperſed through the volume a great number of valuable obſervations, ſuch as might be expected from the abilities and experience of the ingenious and reſpectable author.

In treating of eryſipelata, occaſioned by contagious miaſmata, he introduces ſome very valuable obſervations on the *ſcarlatina anginoſa*, which prevailed in 1779, and has been ſo well deſcribed by the learned Dr. Withering of Birmingham.

Under the head of inflammatory rheumatiſm, he mentions a remarkable inſtance of the good effects of purging in that diſeaſe.

As a proof that the gout is hereditary, he mentions his having ſeen it inherited by a common labourer, who was the natural ſon of a gentleman.

Dr. Kirkland is of opinion, that all the preparations of mercury are decompoſed in the *primæ viæ*; and that giving ſmall doſes of this remedy adds to its efficacy, becauſe a grain of quickſilver, he obſerves, taken twice a day, very often affects the teeth, if not prevented, in in a very ſhort time; whereas a large quantity paſſes off ſometimes without any remarkable effect. As an inſtance of this, he mentions the caſe of a boy, who ſwallowed three pounds of quickſilver, by miſtake, with his milk-porridge. The boy's maſter, who was a barometer-maker, kept the boy at home, and provided conveniencies for collecting his evacuations, in which he expected the quickſilver to appear: but in this he was miſtaken, for a blueiſh ſmut only, which ſeemed to come from every part of his body, appeared on the ſheets, and he never experienced any particular ſenſations from this enormous doſe of mercury.

